# Reply to M. Dhanushkodi

**DOI:** 10.1200/JGO.2016.006130

**Published:** 2016-07-27

**Authors:** Iya E. Bassey, Okon E. Essien, Rebecca M. Gali, Alphonsus E. Udoh, Uwem O. Akpan, Enakirerhi E. Glen

**Affiliations:** **Iya E. Bassey**, **Okon E. Essien**, **Alphonsus E. Udoh** and **Uwem O. Akpan**, University of Calabar, Calabar; **Rebecca M. Gali**, University of Maiduguri, Maiduguri; and **Enakirerhi E. Glen**, University of Calabar Teaching Hospital, Calabar, Nigeria.

We thank Dhanushkodi^1^ sincerely for his questions regarding our study, “Cardiovascular Disease Risk Factors: How Relevant in African Men with Prostate Cancer Receiving Androgen-Deprivation Therapy?”^2^ In our facility, the gonadotropin-releasing hormone agonist used is goserelin. The dose administered is 3.6 mg every 28 days subcutaneously until the prostate-specific antigen (PSA) level is 0 ng/mL. Then the patient is given a drug holiday and put under observation. Treatment is restarted if relapse occurs. The treatment-naïve groups were younger than the treated patients, which would be expected, because the treated patients have had the disease for longer. There was no difference in the ages of those who underwent orchiectomy and those on hormonal manipulation.

In our study, the patients who underwent surgical androgen-deprivation therapy (ADT) had less incidence of cardiovascular risk factors than those who underwent hormone manipulation alone. The mechanism for this is unclear. However, it could suggest that agents used for hormone manipulation may alter other metabolic pathways apart from that of testosterone that may adversely affect lipid profiles.

Blood pressure was assessed in this study, and there was no significant difference (*P* < .05) in the blood pressures of those receiving ADT and those not receiving ADT.^2^ Our study was cross-sectional, so we did not follow up with those patients receiving ADT to see if they had any cardiovascular events. That is also why we do not have baseline values of glucose, lipid profile, and indices of obesity.

The patients who underwent orchiectomy did not have statistically higher PSA values compared with any of the groups. It was actually the treatment-naïve group who had statistically higher mean PSA values compared with patients who underwent orchiectomy alone or hormone manipulation.^2^
